# Associations of Lactoferrin-Fortified Formula with Infant Growth and Gut Microbiota: A Real-World Observational Study

**DOI:** 10.3390/nu17243896

**Published:** 2025-12-12

**Authors:** Xiaojin Shi, Biao Liu, Wenhui Ye, Xuanjing Qi, Menglu Xi, Shuqi Liu, Qihan Zhu, Lutong Zheng, Ai Zhao

**Affiliations:** 1 Vanke School of Public Health, Tsinghua University, Beijing 100084, China; 2 Inner Mongolia Dairy Technology Research Institute Co., Ltd., Hohhot 010110, China; 3 College of Food Science and Engineering, Changchun University, Changchun 130022, China; 4 Institute for Healthy China, Tsinghua University, Beijing 100084, China

**Keywords:** lactoferrin, infants, growth, gut microbiota, real-world study

## Abstract

Background/Objectives: Lactoferrin, a key bioactive component in human milk, may bridge functional gaps in infant formula; however, its long-term effects on growth and the gut microbiota in term infants remain underexplored, particularly in real-world settings. Methods: This real-world evidence (RWE) study assessed the impact of lactoferrin-fortified formula (LF) on infant growth, the gut microbiota, and feeding tolerance compared with control formula (CF) and exclusive breastfeeding (BF). After propensity score matching (PSM) for maternal education level and infant age, 111 matched Chinese infants (37 per group: LF, CF, and BF; age: 6–12 months) were analyzed. Growth was evaluated using WHO Z-scores (WAZ, LAZ, WLZ, and zBMI). The gut microbiota was profiled via 16S rRNA sequencing (*n* = 81). Feeding challenges were quantified using the Montreal Children’s Hospital Feeding Scale (MCH-FS). Results: The LF group exhibited significantly higher length-for-age Z-scores (LAZ) compared with both the BF and CF groups (*p* < 0.001), indicating superior linear growth. LF infants also showed reduced MCH-FS scores (18.0 vs. 36.2 in CF; *p* < 0.001), signifying fewer feeding difficulties. Gut microbiota analysis revealed enrichment of *Bifidobacterium breve* and butyrate-producing taxa (e.g., *Faecalibacterium* and Ruminococcaceae), higher alpha diversity, and metabolic divergence, involving enhanced lysine fermentation to acetate/butyrate in LF infants, suggesting a higher level of short-chain fatty acid (SCFA) production. Beta diversity analysis demonstrated that the LF microbiota clustered close to BF. Conclusions: Lactoferrin-fortified formula was associated with improved linear growth and feeding tolerance while shaping a healthy gut microbiota, showing similarities to breastfed infants’ microbiota. These findings support LF fortification as a strategy to improve functional outcomes in formula-fed infants.

## 1. Introduction

Infant formula feeding has become increasingly common in China [1,2], yet growing evidence suggests that formula-fed infants often exhibit gut microbiota dysbiosis and impaired growth compared with breastfed counterparts [3,4,5,6,7]. Previous studies highlight that traditional infant formulas lack key bioactive components, such as lactoferrin, which may contribute to these disparities [8,9]. In China, where exclusive breastfeeding rates remain suboptimal, understanding the real-world impact of formula composition on infant health is critical.

Lactoferrin, a multifunctional glycoprotein abundant in human milk, plays a crucial role in the development of infants by regulating the intestinal microbiota and promoting growth [9,10]. It shows antimicrobial activity against pathogens while selectively supporting beneficial bacteria such as *Bifidobacterium* [11,12]. Lactoferrin also improves intestinal barrier function and regulates immune responses, which lowers infection-related morbidity rates [13]. In preterm infants, lactoferrin supplements have been shown to lower sepsis rates and improve gut microbial balance [14]. Lactoferrin was found to facilitate nutrient absorption, particularly iron, manganese, and zinc [12], which contributed to improving the growth of infants [15,16,17]. Therefore, adding lactoferrin to infant formula to better mimic human milk composition may contribute to improving health outcomes in formula-fed infants.

However, the long-term effects of lactoferrin on term infants’ growth and microbiota in real-world settings remain underexplored. Although several interesting clinical findings have arisen from controlled trials [18,19], extant research primarily focuses on preterm infants or short-term outcomes [20,21], with limited longitudinal data regarding term infants. Moreover, the interplay between lactoferrin supplementation, the gut microbiota, and infant growth in diverse populations requires further exploration [19].

Therefore, this real-world study aimed to analyze the effects of lactoferrin-fortified infant formula on growth parameters and gut microbiota composition in Chinese infants. We hope to bridge the gap between controlled trials and practical applications by analyzing data from infants with sustained lactoferrin supplementation. Our findings will provide useful insights into optimizing infant nutrition to replicate the advantages of breastfeeding, ultimately supporting healthier growth and microbiota development in formula-fed infants.

## 2. Materials and Methods

### 2.1. Study Design and Setting

This real-world evidence (RWE) study was an open-label investigation conducted in China. We used a hybrid recruitment strategy combining online and offline approaches to evaluate the effects of lactoferrin-fortified infant formula (LF) on growth, feeding difficulties, and the gut microbiota compared with control formula (CF) and exclusive breastfeeding (BF). LF was fortified with additional lactoferrin compared with CF. Detailed ingredient comparisons are provided in Section B of the Supplementary Materials. Given the inherent confounding biases in observational studies, propensity score matching (PSM) was applied to minimize demographic and obstetric heterogeneity between the feeding groups, thereby enhancing the validity of comparative analyses in this real-world setting.

### 2.2. Participants

Infants aged 6–12 months were enrolled through two primary channels: (1) The LF and CF groups were recruited online (mothers club), with infants exclusively fed LF (lactoferrin concentrations: 470 mg/100 g for 0–6 months, and 410 mg/100 g for 6–12 months) or CF (lactoferrin concentration: 0 mg/100 g) starting from day 15 after birth until enrollment. (2) The BF group was recruited from two maternal and child hospitals, matching the same age stratification and exclusively breastfed for the first 4 months. Healthy term infants (gestational age ≥ 37 weeks; Apgar score ≥ 8) with verified exclusive feeding histories at enrollment and no prior formula exposure were recruited. Infants with congenital or metabolic diseases, a history of exposure to immunosuppressants, antibiotics, and prebiotics within 4 weeks before enrollment, or mixed feeding during the intervention period were excluded. Infants in the BF group were exclusively breastfed for the first 4 months and were still receiving breast milk as a primary source of nutrition at the time of enrollment (6–12 months), though most had also introduced complementary foods, consistent with standard feeding practices at this age.

### 2.3. Sample Size and Recruitment

The minimum sample size required to detect significant differences in infant growth between the three groups was calculated to be 159 participants in total, with 53 participants per group, assuming a significance level of 0.05 and a power of 90%. This calculation was based on the maximum mean difference of 337.1 g and an average standard deviation of 415.46 g, derived from the weight change data of two groups in previous studies and a hypothesized third group [22].

**Figure 1:**
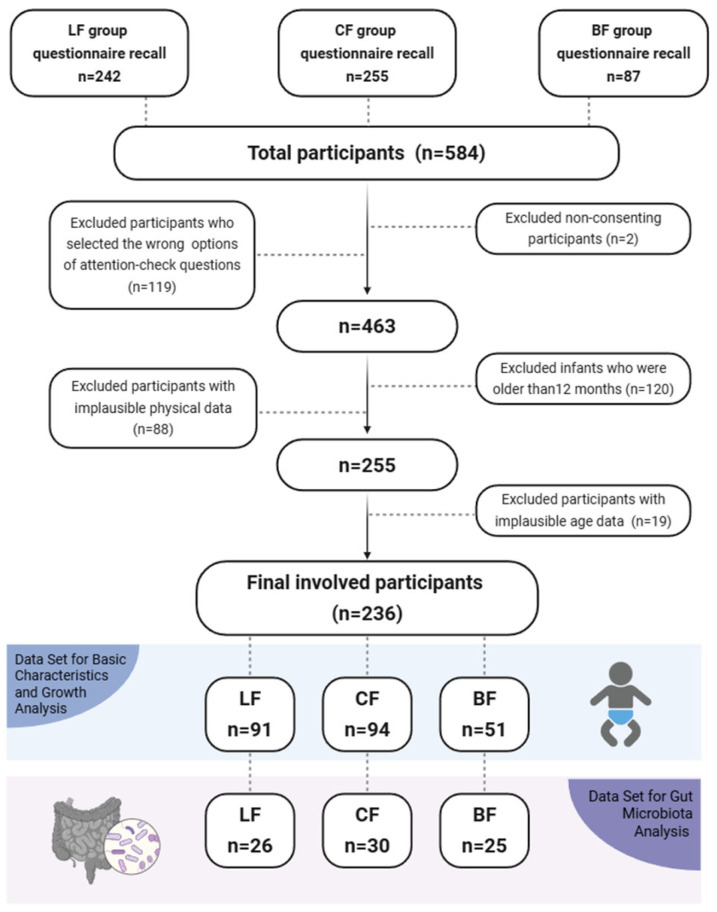
Flowchart of data selection.

A total of 584 infants were initially enrolled (Figure 1). After a multi-step screening process that included administration of a structured digital questionnaire (assessing feeding history, medication use, and health status) and eligibility verification, 236 infants qualified for pre-PSM analysis. Attrition during screening was due to non-consent (*n* = 2), incorrect responses to attention-check questions in the questionnaire (*n* = 119), infants older than 12 months (*n* = 120), implausible physical data (*n* = 88), and implausible age data (*n* = 19). Subsequently, PSM generated a balanced cohort of 111 infants (37 per group: LF, CF, and BF) for final comparisons. Given that our study ultimately included 236 participants, the achieved sample size was sufficient to meet the statistical requirements for detecting significant differences in infant growth between the groups.

### 2.4. Propensity Score Matching (PSM)

We performed propensity score matching (PSM) across the three feeding groups to address significant baseline imbalances in infant age and maternal education level (Table S1). The matching employed a 1:1:1 nearest neighbor algorithm with a caliper width of 0.2 standard deviations of the propensity score. During implementation, the matching process minimized group differences via Mahalanobis distance optimization within propensity score calipers, resulting in reduced standardized mean differences (SMDs) for key covariates post-PSM. This mitigated major confounding effects while preserving real-world sociodemographic variability.

### 2.5. Data Collection

From August 2024 to November 2024, we collected the basic characteristics of the infants and their parents via a structured, self-reported questionnaire administered via an online platform (Wenjuanxing, Beijing, China). The questionnaire included five sections: (1) The maternal demographics section included age, education level, residential location, pet ownership, and household size. (2) The medical history section included maternal and paternal allergy history. (3) The pregnancy and anthropometric indicators section included maternal height, pre-pregnancy weight, weight at delivery, and gestational age. (4) The reproductive history section included gravidity, parity, and delivery type. (5) The infant characteristics section included infant age, birth weight, birth length, infant weight at recruitment, infant length at recruitment, and feeding challenges. Infant anthropometric data (length and weight) and feeding information were collected by asking caregivers to transcribe the relevant records from the standard “Infant Health Handbook”—a nationally unified child health record used across maternal and child health institutions in China. This handbook documents clinical measurements (including length and weight) conducted by healthcare professionals during routine check-ups, as well as feeding-related information recorded in the maternal and child health care records, all of which are transcribed by caregivers for data collection.

According to the information gathered, the WHO Child Growth Standards were used to compute weight-for-age Z-scores (WAZ), length-for-age Z-scores (LAZ), weight-for-length Z-scores (WLZ), and BMI-for-age Z-scores (zBMI). Gestational weight gain was calculated as delivery weight minus pre-pregnancy weight. Postnatal weight gain was calculated as infant weight at recruitment minus birth weight, and postnatal length gain was calculated as infant length at recruitment minus birth length.

Feeding challenges were evaluated using the Chinese version of the validated Montreal Children’s Hospital Feeding Scale (MCH-FS), which included 14 items [23]. This scale assesses key aspects, such as caregiver perceptions and concerns, infant appetite, feeding rhythm and timing, mealtime behavior, food acceptance and handling, caregiver feeding interactions, eating skills, growth outcomes, and socio-emotional impacts. Each item uses a 7-point Likert scale. Thus, the total score ranges from 14 (minimal to no challenges) to 98 (severe, pervasive difficulties), with higher scores reliably indicating more significant challenges, such as disrupted routines, infant distress, or increased caregiver burden. Ultimately, this approach retains the validated structure of the original MCH-FS while adding crucial granularity to capture the nuanced, real-world experiences of infant feeding, including behavioral, emotional, and relational impacts.

### 2.6. Gut Microbiota 16S rRNA Gene Sequencing

Within the pre-PSM population, we additionally collected fecal samples from 81 infants for 16S rRNA gene sequencing. Fecal samples were collected in sterile specimen tubes, immediately flash-frozen in liquid nitrogen, and stored at −80 °C until analysis. Microbial DNA was extracted from the fecal samples, and the V3–V4 region of bacterial 16S rRNA gene was amplified. Libraries were prepared following standard Illumina protocols and sequenced on the NovaSeq 6000 platform (2 × 250 bp; ~45,000 reads per sample). Bioinformatics processing was performed using QIIME2 (v2022.02), including quality filtering, amplicon sequence variant (ASV) generation via DADA2, chimera removal, and taxonomic classification against SILVA 138.1. This process resulted in a final dataset comprising 81 samples, with an average sequencing depth of 37.33 million high-quality bases per sample (range: 21.14 to 58.66 million). This corresponds to an average of 90,019 quality-filtered, non-chimeric reads retained per sample (range: 51,608 to 141,573; median: 89,608), representing an average retention rate of 84.8% from the raw reads. To ensure equitable comparison of microbial community richness and diversity across samples, all subsequent alpha-diversity analyses were performed on data that were rarefied (subsampled without replacement) to a uniform depth of 46,873 sequences per sample. All samples were processed in a single batch to minimize technical batch effects. Detailed methodological descriptions are provided in Section C of the Supplementary Materials.

### 2.7. Statistical Analysis

Statistical analyses were performed using R 4.3.1 and QIIME2 v2022.02. PSM was applied to address baseline heterogeneity between the feeding groups (LF/CF/BF) using nearest neighbor matching. The post-PSM balance was validated via standardized mean differences (SMD < 0.1) and intergroup *p*-values.

Comparative analyses were conducted in both pre- and post-PSM datasets. Continuous variables were compared using one-way ANOVA (for normally distributed data) or Kruskal–Wallis tests (for non-parametric data). Categorical variables were analyzed using chi-square tests or Fisher’s exact tests. Significant ANOVA results (*p* < 0.05) underwent post hoc pairwise comparisons (Tukey’s HSD) with notation: (a) LF vs. CF, (b) LF vs. BF, and (c) CF vs. BF. Age-stratified growth comparisons were also conducted, visualized using grouped box plots.

Growth indicators (WAZ, LAZ, WLZ, and zBMI) were derived using the anthro package (version 0.9.0) in R. The anthro_zscores() function was applied, which requires the exact age in days for each infant as a mandatory input parameter (age_in_days). This age was calculated precisely from the infant’s date of birth and the date of data collection. Growth indicators (WAZ, LAZ, WLZ, and zBMI) were subjected to multivariable linear regression analysis to quantify associations with feeding groups. Model 0 served as an unadjusted model. Model 1 adjusted for maternal age, household size, maternal education level, and maternal allergy history to address potential confounding. Model 2 further incorporated pre-pregnancy weight, maternal height, delivery weight, gravidity, parity, and delivery type as covariates, thereby extending adjustment to obstetric and anthropometric factors. All regression results were visualized using forest plots, depicting β-coefficients (linear regression) alongside their 95% confidence intervals (95% CIs) to illustrate effect magnitudes and precision.

Gut microbiota analysis was conducted on 81 infants, encompassing multiple analytical dimensions. Subsequent analyses included alpha diversity (Shannon, Chao1, Simpson, and observed features) and beta diversity metrics (Bray–Curtis distance). Alpha diversity indices were used to assess group differences, with statistical significance tested via the Kruskal–Wallis test followed by Dunn’s post hoc analysis. To evaluate significant differences in beta diversity, group differences along the primary PCoA axes (PC1 and PC2, based on Bray–Curtis distance) were tested using either ANOVA (for normally distributed data) or the Kruskal–Wallis test (for non-normal data), as determined by normality tests. For taxonomic profiling, microbial biomarkers exhibiting group-specific enrichment were identified using linear discriminant analysis effect size (LEfSe) with a stringent linear discriminant analysis (LDA) score threshold of >4 and *p*-value < 0.05. The functional potential of microbial communities was inferred via phylogenetic investigation of communities by reconstruction of unobserved states (PICRUSt2) to predict Kyoto Encyclopedia of Genes and Genomes (KEGG) metabolic pathways.

### 2.8. Ethical Considerations

Informed consent was obtained from guardians of all infants in the LF and CF groups before completing the online questionnaire by clicking the “Agree” option to confirm willingness to participate voluntarily in this study. Written informed consent was obtained from all BF infants’ guardians before data collection. The study protocol was approved by the Tsinghua University Science and Technology Ethics Committee (Medicine) (approval number: THU01-20240110) and conducted in accordance with the Declaration of Helsinki.

## 3. Results

### 3.1. Subject Disposition and Demographics

In this study, 584 infants were enrolled, with 242 LF, 255 CF, and 87 BF infants (Figure 1). The numbers of participants included in the overall analysis were 91 (LF), 94 (CF), and 51 (BF), respectively. The demographics and baseline characteristics of infants in the overall pre-PSM analysis set are presented in Table S1.

Table S1 presents the mean maternal age at delivery in the LF, CF, and BF groups as 31.1, 27.3, and 31.7 years, respectively. Compared with mothers of infants in the BF and LF groups, mothers of infants in the CF group were more likely to be primigravid and primiparous; have a vaginal delivery; be younger; have a smaller household size; be taller; have lower gestational weight gain; and reside in northern China. Regarding maternal education status, the BF group demonstrated significantly higher levels than the CF and LF groups. There were no significant differences observed in pet ownership across the groups.

PSM was conducted on the most imbalanced and clinically relevant variables (infant age and maternal education level), identified via intergroup heterogeneity analysis. The matched dataset comprised 37 infants from each group (LF, CF, and BF). The demographics and baseline characteristics of the infants included in the post-PSM dataset are shown in Table 1. Post-PSM analyses revealed that intergroup disparities in infant age shifted from statistically significant (pre-PSM: *p* = 0.01; SMD = 0.299) to non-significant (post-PSM: *p* = 0.964; SMD = 0.042), while maternal education levels continued to show significant differences, although there was a measurable reduction in standardized mean differences (SMDs) from 1.017 to 0.825. Despite PSM, several baseline characteristics remained significantly different across groups. For instance, the difference in parental allergy history across all three groups was diminished after matching. Other characteristics exhibited stability comparable with pre-PSM distributions, with no substantial changes observed in intergroup variability (Table 1).

### 3.2. Growth

Post-PSM analysis of infant growth variables (Table 2) revealed that the CF group had a significantly higher birth weight than the other groups. No significant differences were observed between the three groups in birth length, weight-for-age Z-scores (WAZs), or postnatal length gain. The LF group had significantly lower weight-for-length Z-scores (WLZs) and BMI-for-age Z-scores (zBMIs) than the BF group. The LF group demonstrated superior linear growth outcomes, with significantly higher length/height-for-age Z-scores (LAZs) relative to the other two groups.

Based on the age distribution of infants (Table S3), those aged 11 and 12 months were merged into a ≥11-month subgroup for subsequent analyses due to the small sample size of 12-month-old infants.

**Figure 2:**
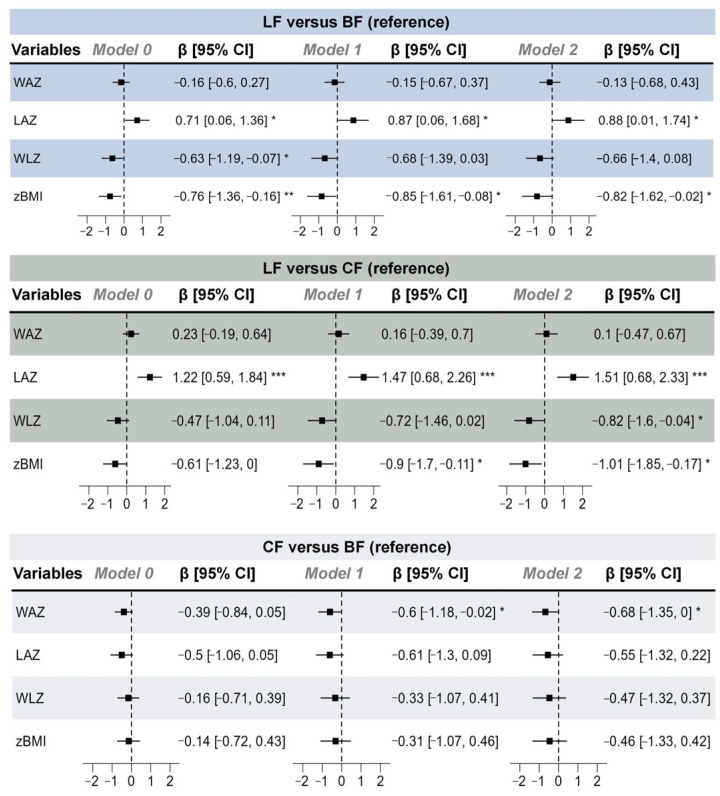
Forest plot of post-propensity score matching (PSM) linear regression results between different feeding groups and infant growth indicators. Model 0 served as an unadjusted model; model 1 adjusted for maternal age, household size, maternal education level, and maternal history of allergies; and model 2 further adjusted for pre-pregnancy weight, maternal height, delivery weight, gravidity, parity, and delivery type. *n* (LF) = 37, *n* (CF) = 37, and *n* (BF) = 37. * *p* < 0.05, ** *p* < 0.01, *** *p* < 0.001.

In the post-PSM data, the LF group demonstrated significant advantages in both the older subgroup (≥11 months) and younger age subgroups, with significantly higher LAZs than those of the other groups (Figure S2). In contrast, the weight-related variables (postnatal weight gain, WAZ, WLZ, and zBMI) of the LF groups were similar to those of the BF group between different age subgroups. The forest plot of post-PSM linear regression results between different feeding groups and infant growth variables (Figure 2) showed that the LF group had significantly higher LAZ values than the other groups in all models.

### 3.3. Feeding Challenges

In the pre-PSM analysis (Table S2), the LF group had significantly lower MCH-FS scores (20.2) than the BF group (28.4) and CF group (35.9), with significant differences observed across all pairwise comparisons. Furthermore, the LF group maintained its superiority in post-PSM analyses (Table 2). Collectively, these findings indicate that the LF group had substantially fewer caregiver-reported feeding challenges than the other groups.

### 3.4. Infant Fecal Microbiota

**Figure 3:**
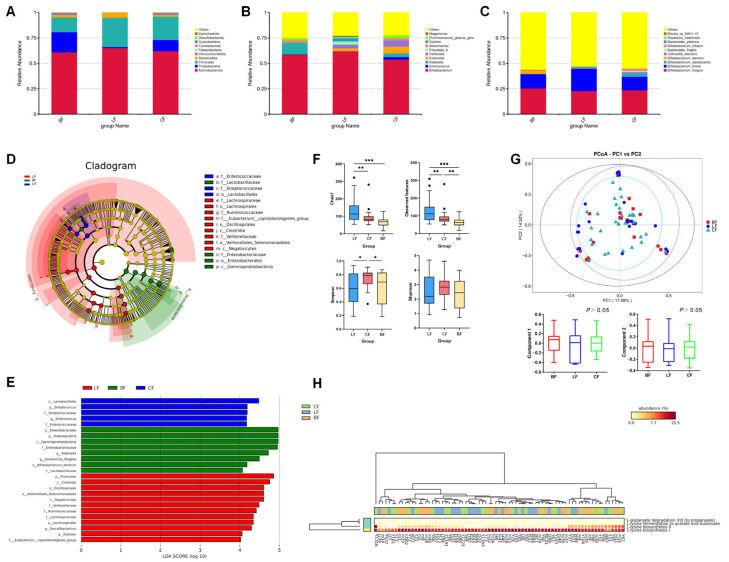
Relative abundances of infant fecal microbiota at the (**A**) phylum, (**B**) genus, and (**C**) species levels. (**D**,**E**) LEfSe analysis between different groups. Analysis of (**F**) α-diversity and (**G**) β-diversity of fecal microorganisms. (**H**) Clustering tree based on pathway database group community difference analysis. LF: lactoferrin-fortified formula; CF: control formula; BF: breastfeeding; *n* (LF) = 26, *n* (CF) = 30, and *n* (BF) = 25. * *p* < 0.05, ** *p* < 0.01, *** *p* < 0.001.

We further collected fecal samples from 81 infants (LF: 26; CF: 30; and BF: 25) and conducted 16S rRNA gene sequencing, which revealed that distinct infant feeding regimens (LF, CF, and BF) profoundly shaped gut microbiota composition, diversity, and metabolic functionality, revealing significant group-specific signatures across multiple taxonomic levels and functional analyses. Analysis of infant fecal microbiota among the three infant groups at three taxonomic levels (phylum, genus, and species) is separately shown in Figure 3. At the phylum level (Figure 3A), the LF group exhibited a higher abundance of Firmicutes (28.68%) compared with the other two groups (BF: 14.41%; CF: 22.83%), while the BF group showed a higher level of Proteobacteria (BF: 19.96%; LF: 1.04%; and CF: 11.05%). At the genus level (Figure 3B), the LF group demonstrated the highest relative abundance of *Bifidobacterium* (61.97%) and the lowest abundance of *Akkermansi*a (0.21%) among the groups (BF: 58.34% and CF: 53.76%; BF: 2.20% and CF: 2.08%). The CF group displayed the highest proportion of *Enterococcus* (CF: 2.62%; LF: 0.02%; and BF: 0.39%), whereas the BF group was characterized by a predominance of *Klebsiella* (BF: 11.29%; LF: 0.19%; and CF: 3.50%). Species-specific analysis (Figure 3C) showed that *Bifidobacterium breve* was significantly enriched in the LF group (LF: 22.28%; CF: 13.40%; and BF: 14.15%). The CF group showed higher abundances of *Bifidobacterium adolescentis* (2.85%) and *Bifidobacterium bifidum* (2.19%) compared with the other groups (BF: 0.00% and LF: 0.14%; BF: 0.71% and LF: 0.56%). In contrast, the BF group exhibited a marked increase in *Bifidobacterium dentium* abundance.

The heatmap (Figure S3) displays the enrichment distribution of the fecal microbiota composition at the phylum, genus, and species levels among different feeding groups. The predominant phyla in the fecal microbiota exhibited group-specific variations (Figure S3A). Proteobacteria and Verrucomicrobiota dominated in the BF group, whereas Bacteroidota and Actinobacteria were prevalent in the LF group, with Gemmatimonadota constituting the primary phylum in the CF group. The main genera, shown in Figure S3B, included *Eubacterium*, *Lactobacillus*, *Klebsiella*, and *Parabacteroides* in the BF group; *Ruminococcus*, *Faecalibacterium*, and *Bifidobacterium* in the LF group; and *Bacteroides* and *Enterococcus* in the CF group. The main species, shown in Figure S3C, included *Bifidobacterium dentium*, *Bifidobacterium longum*, and *Bifidobacterium animalis* in the BF group; *Bifidobacterium breve* and *Lactobacillus mucosae* in the LF group; and *Bifidobacterium adolescentis* and *Bifidobacterium bifidum* in the CF group.

LEfSe analysis (LDA score > 4; *p* < 0.05) revealed significant intergroup differences in microbial biomarkers (Figure 3D,E). Specifically, the BF group demonstrated pronounced enrichment of Proteobacteria, Lactobacillaceae, *Escherichia-Shigella*, *Klebsiella*, and *Bifidobacterium dentium*. In contrast, the CF group was characterized by elevated abundances of Lactobacillales, *Enterococcus*, and *Streptococcus*, whereas the LF group showed dominant representation of Ruminococcaceae, Veillonellaceae, Lachnospiraceae, and *Faecalibacterium*.

Figure 3F showed the alpha diversity indices of infant fecal microbiota, such as observed features, Chao1, Shannon, and Simpson indices. The results demonstrated that the LF group exhibited significantly higher values of both observed features and the Chao1 index compared with the BF and CF groups (*p* < 0.01). In contrast, the CF group showed a higher Simpson index level (*p* < 0.05).

Based on the Bray–Curtis distance, beta diversity analysis revealed that the microbiota composition of the LF and CF groups was not significantly different from that of the BF group (Figure 3G). The principal coordinate components PC1 and PC2, which explained the most variation in the data, accounted for 17.08% and 14.52%, respectively.

Figure 3H illustrates the differential metabolic pathways of the gut microbiota across different feeding groups. The results revealed significant intergroup variations in lysine fermentation pathways to acetate/butyrate and lysine biosynthesis pathways. Specifically, fecal samples from infants in the LF group exhibited significantly higher expression of lysine fermentation pathways producing acetate, butyrate, and propionate, alongside markedly reduced lysine biosynthesis activity, compared with the other groups (*p* < 0.01), which indicated that LF formula might promote the microbial conversion of lysine into acetate, butyrate, and propionate rather than endogenous lysine synthesis.

## 4. Discussion

Infant nutrition serves as a cornerstone for lifelong health, with early feeding practices critically influencing growth trajectories and gastrointestinal development. This real-world study investigated the effects of lactoferrin-fortified formula on infant growth, gut microbiota, and feeding challenges, using propensity score matching (PSM) to mitigate confounding and provide robust evidence for clinical practice. This study demonstrates that LF feeding is associated with superior linear growth (higher LAZ scores) in infants, potentially mediated through its beneficial modulation of gut microbiota. LF feeding shapes a distinctive microbial profile enriched with commensal taxa and enhances metabolic pathways for short-chain fatty acid (SCFA) production, which may contribute to improved nutrient metabolism. Furthermore, LF-fed infants exhibited better feeding tolerance, as evidenced by significantly fewer caregiver-reported feeding difficulties.

### 4.1. Superior Linear Growth with Favorable Adiposity Trajectories

A striking result of our study was the significantly higher LAZ in the LF group, even after adjusting for confounders. Notably, compared with the LF group, the BF group had mothers with higher education levels, and the CF group had younger, taller, and healthier mothers. These differences in maternal characteristics may have led to suboptimal feeding practices, as well as poorer health and growth trends in infants of the LF group [24,25,26,27]. Even though the infants in the LF group had relatively disadvantageous maternal conditions compared with those in the other two groups, their linear growth showed a significant advantage, which highlighted the potential of lactoferrin-fortified formula as a growth-promoting intervention. Some previous studies revealed that lactoferrin fortification enhances linear growth in term infants, possibly via its role in promoting mineral absorption, bone formation, bone mineral density, and strength [28,29]. Furthermore, the sustained LAZ advantage observed in LF infants across all age subgroups (≥11 months and younger) may be supported by mechanistic insights from prior literature. Previous in vitro and animal studies have proposed that lactoferrin could promote bone formation through interactions with pathways such as LRP1, PI3K/Akt, and MAPK, which are involved in osteoblast proliferation and differentiation [28,30,31,32,33,34]. In addition, lactoferrin has been suggested to modulate the vitamin D receptor, a key regulator of bone metabolism [35]. While the present study did not measure these specific pathways, these proposed mechanisms offer a plausible biological framework for our findings. Future research directly investigating these pathways in humans is warranted to confirm their role in mediating the effects of lactoferrin on linear growth.

While LF infants had lower WLZ and zBMI scores than BF infants, these differences were not associated with underweight or growth faltering. Instead, our results suggest a more favorable growth trajectory with reduced adiposity, which is particularly critical given concerns about excessive weight gain in formula-fed infants and its link to childhood obesity. This contrasts with a randomized controlled trial (RCT) reporting increased weight gain in LF infants [36]; however, our real-world design may better reflect clinical practice, where BF infants often receive complementary foods later or have lower energy intake [37]. Additionally, LF was observed to reduce visceral fat accumulation and body weight in both animal models and clinical studies [38,39]. The absence of significant differences in WAZ or postnatal weight gain between the LF and BF groups further supports that LF formula prioritizes healthy growth over rapid weight accumulation.

### 4.2. Lactoferrin Modulates Composition, Diversity, and Metabolic Function

The gut microbiota analysis revealed robust group-specific signatures, with LF feeding driving a gut microbiota profile closer to that of BF than that of CF. At the phylum level, the LF group had a higher Firmicutes abundance compared with BF and CF, a pattern linked to increased SCFA production [40]. Firmicutes include taxa such as *Faecalibacterium* and Ruminococcaceae, which are key producers of butyrate, an SCFA critical for intestinal barrier function, anti-inflammatory effects, and energy metabolism [41]. The LF group also showed enriched *Bifidobacterium breve*, a species associated with reduced intestinal inflammation and improved nutrient absorption and growth in infants [42,43]. This is consistent with the results of a previous study on the prebiotic effects of lactoferrin, which selectively promote beneficial bacteria by inhibiting pathogens such as *Klebsiella*, a pathogen that was found to be more abundant in breast-fed infants [44].

The alpha diversity indices (observed features and Chao1) were significantly higher in the LF group, indicating greater microbial richness, which is associated with rapid infant growth in the first year of life [45]. Although the clustering based on community structure does not imply full functional equivalence, beta diversity analyses (PCoA) indicated that the gut microbiota composition of the LF group was similar to that of the BF group.

According to mechanistic insights from metabolic pathway analysis inferred via PICRUSt2, LF infants had increased activity in lysine fermentation pathways producing short-chain fatty acids (SCFAs), particularly acetate, butyrate, and propionate, while lysine biosynthesis was reduced. This suggests that lactoferrin might promote the microbial conversion of dietary lysine into SCFAs rather than endogenous synthesis. In addition, SCFAs have been shown to stimulate growth hormone release via the gut–brain axis and improve nutrient uptake by modulating intestinal permeability [46,47], which may explain the positive correlation between LF infants’ microbiota effects and superior linear growth. While these findings suggest a potential role for SCFAs in mediating the observed growth benefits, we did not directly measure SCFA concentrations in stool samples. Therefore, our hypothesis regarding SCFA mediation remains speculative and requires direct metabolite data for validation. Thus, this proposed mechanistic model, based on predictive functional profiling, requires validation through direct metagenomic and metabolomic assays in future studies.

Additionally, Proteobacteria had a higher abundance in BF infants compared with LF and CF. Proteobacteria are often associated with intestinal inflammation in infants, but their role in BF is complex, as breast milk contains antibodies that may mitigate their pathogenic effects [48]. The low Proteobacteria abundance in LF infants may reflect lactoferrin’s direct antimicrobial activity against Gram-negative bacteria [49], which is a potential advantage in environments with high pathogen exposure and no protection from breast milk antibodies. However, the findings from the gut microbiota analysis, while suggestive, should be interpreted with caution as they derive from an imbalanced sample and may be influenced by confounding factors, such as infant age. Future studies with larger, prospectively matched cohorts are needed to confirm these associations.

### 4.3. Lactoferrin Reduces Caregiver Burden

The LF infants’ consistently lower MCH-FS scores in both pre- and post-PSM indicate fewer feeding challenges and fewer common concerns in formula-fed infants, such as fussiness, vomiting, or constipation. Furthermore, these improvements in infant comfort represent an additional benefit of lactoferrin that is relatively independent of growth promotion. This may be attributed to the multifunctional benefits of lactoferrin, as it inhibits pathogenic bacteria such as *Escherichia-Shigella*, and reduces inflammation, all of which contribute to improved gastrointestinal tolerance [50]. For caregivers, reduced feeding challenges can alleviate stress and improve feeding adherence, which is crucial in infant health and development. This finding is particularly relevant for populations where breastfeeding is difficult, as it highlights the potential of LF to support both infant and caregiver well-being.

### 4.4. Strengths and Limitations

This study provides real-world evidence of the impact of lactoferrin-fortified formula on infant growth trajectories and gut microbiota composition, a combination that is less frequently investigated outside controlled trials. We utilized rigorous propensity score matching to minimize confounding and ensure robust comparisons. Critically, we extended beyond observational associations by preliminarily exploring the potential mechanisms linking lactoferrin fortification to enhanced linear growth via modulation of beneficial taxa (*Bifidobacterium breve*) and SCFA-producing metabolic pathways. However, several limitations of this study warrant consideration. First, the observational real-world design precludes causal inference between lactoferrin fortification and outcomes. Additionally, both covariates and growth outcomes relied on caregiver-reported data, introducing potential recall and misclassification bias. Future research could therefore incorporate direct clinical assessments to mitigate these biases and enhance the robustness of study findings. Moreover, the observed associations cannot be attributed solely to lactoferrin, as the LF and CF differed in several nutritional components besides lactoferrin (e.g., DHA, ARA, OPO, and prebiotics). Despite PSM, potential residual confounding may have persisted. In addition, the sample size post-PSM (37 per group) and for gut microbiota analysis (81 infants) were relatively small, limiting the power to detect subtle differences. In addition, this study relied on PICRUSt2 for functional prediction and lacked direct SCFA measurement. These inferred functional insights warrant cautious interpretation and highlight the need for direct metabolomic validation in future investigations. Finally, our study focused on a Chinese population; thus, research in other populations is needed to evaluate the generalizability of growth and microbiota effects, as well as their potential links to later-life health.

## 5. Conclusions

In conclusion, our study adds to the growing body of evidence supporting lactoferrin-fortified formula as a beneficial option for infant feeding. The superior linear growth, favorable gut microbiota profile, and reduced feeding challenges observed in LF-fed infants support the inclusion of lactoferrin in infant formula as a strategy to promote healthy growth and gut development when exclusive breastfeeding cannot be achieved. Future research should include larger, more diverse cohorts; collect detailed data on maternal diet and feeding practices; conduct long-term follow-up to assess outcomes, such as allergy risk or cognitive development; and investigate the underlying mechanisms linking lactoferrin, microbiota, and growth.

## Figures and Tables

**Table 1:** Post-propensity score matching (PSM) analysis of demographic characteristics, parental health, and obstetric status across 3 feeding groups.

Variables	Overall (*n* = 111)	LF (*n* = 37)	CF (*n* = 37)	BF (*n* = 37)	*p*
Maternal ages, mean (SD), year ^a,c^	30.1 (4.6)	31.5 (4.7)	27.1 (3.3)	31.7 (4.1)	<0.001
Household size, mean (SD) ^a,c^	4.2 (1.2)	4.4 (1.0)	3.7 (1.0)	4.4 (1.4)	0.014
Residential location, *n* (%) ^a,b^					<0.001
Northern China	25 (23)	0 (0)	13 (35)	12 (32)	
Southern China	86 (77)	37 (100)	24 (65)	25 (68)	
Maternal education levels, *n* (%) ^b,c^					<0.001
Junior high school and below	55 (50)	28 (76)	19 (51)	8 (22)	
High school/vocational high school/vocational secondary school/vocational college	51 (46)	8 (22)	16 (43)	27 (73)	
Bachelor’s degree or above	5 (4.5)	1 (2.7)	2 (5.4)	2 (5.4)	
Pet ownership, *n* (%)					0.9
Yes	6 (5.4)	1 (2.7)	3 (8.1)	2 (5.4)	
No	105 (95)	36 (97)	34 (92)	35 (95)	
Maternal allergy history, *n* (%) ^b^					0.016
Yes	6 (5.4)	0 (0)	1 (2.7)	5 (14)	
No	104 (94)	37 (100)	36 (97)	31 (84)	
Unclear	1 (0.9)	0 (0)	0 (0)	1 (2.7)	
Paternal allergy history, *n* (%)					0.056
Yes	7 (6.3)	1 (2.7)	1 (2.7)	5 (14)	
No	102 (92)	36 (97)	36 (97)	30 (81)	
Unclear	2 (1.8)	0 (0)	0 (0)	2 (5.4)	
Maternal height, mean (SD), cm ^a,c^	162.1 (4.9)	159.9 (4.7)	165.0 (3.4)	161.4 (5.1)	<0.001
Pre-pregnancy weight, mean (SD), kg ^b^	55.0 (7.4)	53.3 (5.8)	53.8 (6.2)	57.9 (9.1)	0.046
Maternal weight at delivery, mean (SD), kg ^b,c^	67.0 (7.8)	66.0 (6.2)	64.4 (7.5)	70.6 (8.2)	0.003
Gestational weight gain, mean (SD), kg ^a,c^	12.0 (4.6)	12.8 (3.8)	10.6 (3.6)	12.7 (5.8)	0.019
Gestational age, mean (SD), week ^a,c^	39.0 (1.3)	38.9 (0.6)	39.4 (1.1)	38.7 (1.9)	0.037
Gravidity, *n* (%) ^a,c^					0.009
Primigravida	71 (64)	20 (54)	31 (84)	20 (54)	
Multigravida	40 (36)	17 (46)	6 (16)	17 (46)	
Parity, *n* (%) ^a,c^					0.002
Primipara	78 (70)	21 (57)	34 (92)	23 (62)	
Multipara	33 (30)	16 (43)	3 (8.1)	14 (38)	
Types of delivery, *n* (%) ^a,c^					0.003
Vaginal delivery	65 (59)	18 (49)	30 (81)	17 (46)	
Cesarean delivery	46 (41)	19 (51)	7 (19)	20 (54)	

In the pairwise comparisons of post-PSM data, a statistically significant difference (*p* < 0.05) was observed between the ^a^ LF and CF groups, ^b^ LF and BF groups, and ^c^ CF and BF groups.

**Table 2:** Post-propensity score matching (PSM) analysis of growth and feeding challenge metrics in infants across the 3 feeding groups.

Variables	Overall (*n* = 111)	LF (*n* = 37)	CF (*n* = 37)	BF (*n* = 37)	*p*
Birth weight, mean (SD), kg ^a^	3.3 (0.3)	3.2 (0.3)	3.4 (0.3)	3.3 (0.4)	0.02
Birth length, mean (SD), cm	49.8 (3.1)	50.4 (2.7)	49.0 (4.0)	49.9 (2.3)	0.3
WAZ, mean (SD)	0.3 (0.9)	0.3 (0.9)	0.1 (0.9)	0.5 (1.0)	0.2
LAZ, mean (SD) ^a,c^	0.4 (1.4)	1.1 (1.5)	−0.2 (1.1)	0.3 (1.3)	<0.001
WLZ, mean (SD) ^b^	0.2 (1.2)	−0.1 (1.3)	0.3 (1.2)	0.5 (1.2)	0.069
zBMI, mean (SD) ^b^	0.1 (1.3)	−0.3 (1.4)	0.3 (1.3)	0.4 (1.2)	0.053
Postnatal length gain, mean (SD), cm	21.7 (4.0)	22.6 (4.2)	21.2 (3.5)	21.4 (4.0)	0.3
Postnatal weight gain, mean (SD), kg ^a^	5.0 (1.7)	5.6 (1.2)	4.6 (1.6)	5.0 (2.0)	0.073
MCH-FS score, mean (SD) ^a,b,c^	27.0 (13.3)	18.0 (9.7)	36.2 (11.3)	26.6 (12.2)	<0.001

In the pairwise comparisons of post-PSM data, a statistically significant difference (*p* < 0.05) was observed between the ^a^ LF and CF groups, ^b^ LF and BF groups, and ^c^ CF and BF groups.

## Data Availability

The datasets generated and analyzed during the current study are not publicly available because informed consent was not obtained for data sharing. However, the data are available from the authors upon reasonable request.

## Supplementary Materials

The following supporting information can be downloaded from https://www.mdpi.com/article/10.3390/nu17243896/s1. Figure S1: Box plots of pre-PSM comparative distributions for (a) postnatal weight gain, (b) postnatal length gain, (c) weight-for-age Z-score (WAZ), (d) length-for-age Z-score (LAZ), (e) weight-for-length Z-score (WLZ), and (f) BMI-for-age Z-score (zBMI). Figure S2: Box plots of post-PSM comparative distributions for (a) postnatal weight gain, (b) postnatal length gain, (c) weight-for-age Z-score (WAZ), (d) length-for-age Z-score (LAZ), (e) weight-for-length Z-score (WLZ), and (f) BMI-for-age Z-score (zBMI). Figure S3: Comparative heat map of infant fecal microbial communities at the (A) phylum, (B) genus, and (C) species levels in different feeding groups. Figure S4: Love plot for alternative propensity score matching strategies. Table S1: Comparative analysis of demographic characteristics, parental health, and obstetric profiles across 3 feeding groups, pre- and post-propensity score matching (PSM). Table S2: Comparative analysis of growth and feeding challenge metrics in infants across 3 feeding groups, pre- and post- propensity score matching (PSM). Table S3: Monthly age distribution of infants across 3 feeding groups, pre- and post- propensity score matching (PSM). Table S4: Ingredient comparisons of lactoferrin-fortified infant formula (LF) and control formula (CF). Table S5: Summary of Data Processing.

## Abbreviations

The following abbreviations were used in this manuscript:

LF	Lactoferrin-fortified formula
CF	Control formula
BF	Breastfeeding
RWE	Real-world evidence
MCH-FS	Montreal Children’s Hospital Feeding Scale
LAZ	Length-for-age Z-score
WAZ	Weight-for-age Z-score
WLZ	Weight-for-length Z-score
zBMI	BMI-for-age Z-score
PSM	Propensity score matching
SCFA	Short-chain fatty acid
PCoA	Principal coordinate analysis
LEfSe	Linear discriminant analysis effect size
LDA	Linear discriminant analysis
PICRUSt2	Phylogenetic investigation of communities by reconstruction of unobserved states
KEGG	Kyoto Encyclopedia of Genes and Genomes
