# Supplementary material for: Associations of Lactoferrin-Fortified Formula with Infant Growth and Gut Microbiota: A Real-World Observational Study
Source: Nutrients. 2025 Dec 12;17(24):3896. doi: 10.3390/nu17243896 (PMC12736245; doi:10.3390/nu17243896)
Supplement: Supplementary file 1 [file nutrients-17-03896-s001.zip › nutrients-3986820-supplementary.pdf]

Supplementary material

Section A

Table S1 Comparative analysis of demographic characteristics, parental health, and obstetric profiles across 3 feeding groups pre- and post-propensity score matching (PSM)

Variables	Pre-PSM					Post-PSM				
	Overall (n=236)	LF (n=91)	CF (n=94)	BF (n=51)	p	Overall (n=111)	LF (n=37)	CF (n=37)	BF (n=37)	p
Maternal ages, mean (SD), Year [A,C,a,c]	29.7 (4.4)	31.1 (4.6)	27.3 (2.9)	31.7 (4.2)	<0.001	30.1 (4.6)	31.5 (4.7)	27.1 (3.3)	31.7 (4.1)	<0.001
Household size, mean (SD) [A,C,a,c]	4.1 (1.1)	4.3 (1.0)	3.7 (0.9)	4.3 (1.3)	<0.001	4.2 (1.2)	4.4 (1.0)	3.7 (1.0)	4.4 (1.4)	0.014
Residential location, n (%) [A,a,b]					0.006					<0.001
Northern China	73 (31)	18 (20)	39 (41)	16 (31)		25 (23)	0 (0)	13 (35)	12 (32)	
Southern China	163 (69)	73 (80)	55 (59)	35 (69)		86 (77)	37 (100)	24 (65)	25 (68)	
Maternal education levels, n (%) [A,B,C,b,c]					<0.001					<0.001
Junior high school and below	100 (42)	62 (68)	30 (32)	8 (16)		55 (50)	28 (76)	19 (51)	8 (22)	
High school/vocational high school/vocational secondary school/vocational college	116 (49)	27 (30)	62 (66)	27 (53)		51 (46)	8 (22)	16 (43)	27 (73)	
Bachelor degree or above	20 (8.5)	2 (2.2)	2 (2.1)	16 (31)		5 (4.5)	1 (2.7)	2 (5.4)	2 (5.4)	
Pet ownership, n (%)					0.9					0.9
Yes	17 (7.2)	6 (6.6)	8 (8.5)	3 (5.9)		6 (5.4)	1 (2.7)	3 (8.1)	2 (5.4)	
No	219 (93)	85 (93)	86 (91)	48 (94)		105 (95)	36 (97)	34 (92)	35 (95)	
Maternal allergy history, n (%) [C,b]					0.006					0.016
Yes	11 (4.7)	4 (4.4)	1 (1.1)	6 (12)		6 (5.4)	0 (0)	1 (2.7)	5 (14)	
No	222 (94)	86 (95)	93 (99)	43 (84)		104 (94)	37 (100)	36 (97)	31 (84)	
Unclear	3 (1.3)	1 (1.1)	0 (0)	2 (3.9)		1 (0.9)	0 (0)	0 (0)	1 (2.7)	
Paternal allergy history, n (%) [C]					0.019					0.056
Yes	11 (4.7)	4 (4.4)	2 (2.1)	5 (9.8)		7 (6.3)	1 (2.7)	1 (2.7)	5 (14)	
No	221 (94)	86 (95)	92 (98)	43 (84)		102 (92)	36 (97)	36 (97)	30 (81)	
Unclear	4 (1.7)	1 (1.1)	0 (0)	3 (5.9)		2 (1.8)	0 (0)	0 (0)	2 (5.4)	
Maternal height, mean (SD), cm [A,C,a,c]	162.2 (4.9)	160.0 (4.4)	165.0 (3.9)	161.1 (4.9)	<0.001	162.1 (4.9)	159.9 (4.7)	165.0 (3.4)	161.4 (5.1)	<0.001
Pre-pregnancy weight, mean (SD), Kg [b]	54.9 (7.1)	54.8 (6.6)	54.0 (6.4)	57.0 (8.9)	0.2	55.0 (7.4)	53.3 (5.8)	53.8 (6.2)	57.9 (9.1)	0.046
Maternal weight at delivery, MEAN (SD), Kg [C,b,c]	67.5 (8.3)	68.0 (8.4)	65.8 (7.8)	69.6 (8.4)	0.021	67.0 (7.8)	66.0 (6.2)	64.4 (7.5)	70.6 (8.2)	0.003
Gestational weight gain, mean (SD), Kg [A,a,c]	12.6 (4.9)	13.3 (5.1)	11.9 (4.2)	12.6 (5.8)	0.063	12.0 (4.6)	12.8 (3.8)	10.6 (3.6)	12.7 (5.8)	0.019
Gestational age, mean (SD), Week [A,C,a,c]	39.0 (1.4)	38.9 (0.9)	39.4 (1.5)	38.6 (1.7)	<0.001	39.0 (1.3)	38.9 (0.6)	39.4 (1.1)	38.7 (1.9)	0.037
Gravidity, n (%) [A,C,a,c]					<0.001					0.009
Primigravida	156 (66)	45 (49)	85 (90)	26 (51)		71 (64)	20 (54)	31 (84)	20 (54)	
Multigravida	80 (34)	46 (51)	9 (9.6)	25 (49)		40 (36)	17 (46)	6 (16)	17 (46)	
Parity, n (%) [A,C,a,c]					<0.001					0.002
Primipara	167 (71)	48 (53)	89 (95)	30 (59)		78 (70)	21 (57)	34 (92)	23 (62)	
Multipara	69 (29)	43 (47)	5 (5.3)	21 (41)		33 (30)	16 (43)	3 (8.1)	14 (38)	
Types of Delivery, n (%) [A,C,a,c]					<0.001					0.003
Vaginal Delivery	140 (59)	40 (44)	72 (77)	28 (55)		65 (59)	18 (49)	30 (81)	17 (46)	
Cesarean Delivery	96 (41)	51 (56)	22 (23)	23 (45)		46 (41)	19 (51)	7 (19)	20 (54)	

In the pairwise comparisons of pre-PSM data, a statistically significant difference ($p<0.05$) was observed between the [A] LF and CF groups; [B] LF and BF groups; [C] CF and BF groups.

In the pairwise comparisons of post-PSM data, a statistically significant difference ($p<0.05$) was observed between the [a] LF and CF groups; [b] LF and BF groups; [c] CF and BF groups.

Pre-PSM analysis in infant growth variables (**Table S2**) revealed that the LF group showed significantly lower birth weights compared to the other groups, although their birth lengths were significantly greater than the CF group. There were no significant differences across all three groups in postnatal length gain. Notably, the LF group demonstrated superior linear growth outcomes, with significantly higher length/height for age z-scores (LAZ) and lower postnatal weight gain relative to the other two groups.

Table S2 Comparative analysis of growth and feeding challenge metrics in infants across 3 feeding groups pre- and post- propensity score matching (PSM)

Variables	Pre-PSM					Post-PSM				
	Overall (n=236)	LF (n=91)	CF (n=94)	BF (n=51)	p	Overall (n=111)	LF (n=37)	CF (n=37)	BF (n=37)	p
Birth weight, mean (SD), Kg [A,a]	3.4 (0.4)	3.3 (0.3)	3.4 (0.3)	3.4 (0.5)	0.016	3.3 (0.3)	3.2 (0.3)	3.4 (0.3)	3.3 (0.4)	0.02
Birth length, mean (SD), cm [A,C]	49.5 (3.0)	50.3 (2.4)	48.5 (3.5)	49.9 (2.1)	<0.001	49.8 (3.1)	50.4 (2.7)	49.0 (4.0)	49.9 (2.3)	0.3
WAZ, mean (SD) [A,C]	0.3 (1.0)	0.5 (0.9)	0.1 (1.1)	0.5 (0.9)	0.01	0.3 (0.9)	0.3 (0.9)	0.1 (0.9)	0.5 (1.0)	0.2
LAZ, mean (SD) [A,a,b,c]	0.5 (1.4)	0.9 (1.5)	0.2 (1.3)	0.4 (1.2)	0.002	0.4 (1.4)	1.1 (1.5)	-0.2 (1.1)	0.3 (1.3)	<0.001
WLZ, mean (SD) [b]	0.2 (1.3)	0.2 (1.2)	0.1 (1.4)	0.5 (1.1)	0.3	0.2 (1.2)	-0.1 (1.3)	0.3 (1.2)	0.5 (1.2)	0.069
zBMI, mean (SD) [b]	0.1 (1.4)	0.0 (1.3)	0.0 (1.5)	0.4 (1.1)	0.3	0.1 (1.3)	-0.3 (1.4)	0.3 (1.3)	0.4 (1.2)	0.053
Postnatal length gain, mean (SD), cm	22.3 (4.3)	23.0 (4.6)	22.1 (4.1)	21.4 (4.0)	0.2	21.7 (4.0)	22.6 (4.2)	21.2 (3.5)	21.4 (4.0)	0.3
Postnatal weight gain, mean (SD), Kg [A,B,a]	5.1 (1.6)	5.7 (1.4)	4.7 (1.4)	4.9 (1.8)	<0.001	5.0 (1.7)	5.6 (1.2)	4.6 (1.6)	5.0 (2.0)	0.073
MCH-FS score, mean (SD) [A,B,C,a,b,c]	28.2 (13.1)	20.3 (10.4)	35.7 (11.3)	28.4 (12.3)	<0.001	27.0 (13.3)	18.0 (9.7)	36.2 (11.3)	26.6 (12.2)	<0.001

In the pairwise comparisons of pre-PSM data, a statistically significant difference ($p<0.05$) was observed between the [A] LF and CF groups; [B] LF and BF groups; [C] CF and BF groups.
In the pairwise comparisons of post-PSM data, a statistically significant difference ($p<0.05$) was observed between the [a] LF and CF groups; [b] LF and BF groups; [c] CF and BF groups.

Table S3 Monthly age distribution of infants across 3 feeding groups pre- and post- propensity score matching (PSM)

	**Pre-PSM**				**Post-PSM**			
	**Overall (n=236)**	**LF (n=91)**	**CF (n=94)**	**BF (n=51)**	**Overall (n=111)**	**LF (n=37)**	**CF (n=37)**	**BF (n=37)**
**Age, n (%)**								
**6 months**	45 (19)	13 (14)	15 (16)	17 (33)	29 (26)	5 (14)	10 (27)	14 (38)
**7 months**	57 (24)	15 (16)	36 (38)	6 (12)	16 (14)	10 (27)	3 (8.1)	3 (8.1)
**8 months**	45 (19)	22 (24)	14 (15)	9 (18)	23 (21)	12 (32)	6 (16)	5 (14)
**9 months**	35 (15)	12 (13)	18 (19)	5 (9.8)	15 (14)	3 (8.1)	9 (24)	3 (8.1)
**10 months**	29 (12)	14 (15)	8 (8.5)	7 (14)	16 (14)	2 (5.4)	8 (22)	6 (16)
**≥11 months**	25 (11)	15 (16)	3 (3.2)	7 (14)	12 (11)	5 (14)	1 (2.7)	6 (16)
11 months	19 (8.1)	13 (14)	2 (2.1)	4 (7.8)	8 (7.2)	5 (14)	0 (0)	3 (8.1)
12 months	6 (2.5)	2 (2.2)	1 (1.1)	3 (5.9)	4 (3.6)	0 (0)	1 (2.7)	3 (8.1)

In pre-PSM data, the LF group showed significantly superior linear growth outcomes

across all infant age strata, as illustrated in **Figure S1**. This advantage was particularly pronounced in older infants (≥11 months), where the LF group demonstrated both significantly higher LAZ and postnatal length gain compared to the other two groups. However, there were no significant differences in the weight-related variables (postnatal weight gain, WAZ, WLZ, and zBMI) observed between any groups.

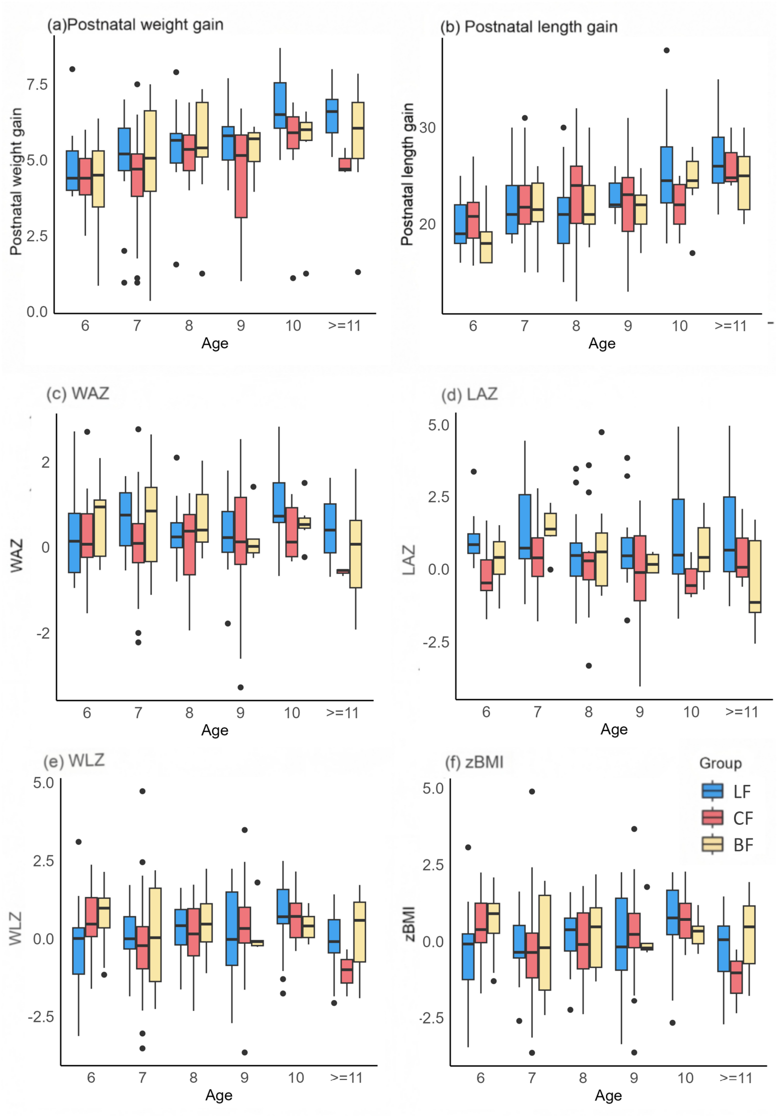

Figure S1. Box plots of pre-PSM comparative distributions for (a) postnatal weight gain, (b) postnatal length gain, (c) weight-for-age Z-score (WAZ), (d) length-for-age Z-score (LAZ), (e) weight-for-length Z-score (WLZ), and (f) BMI-for-age Z-score (zBMI)

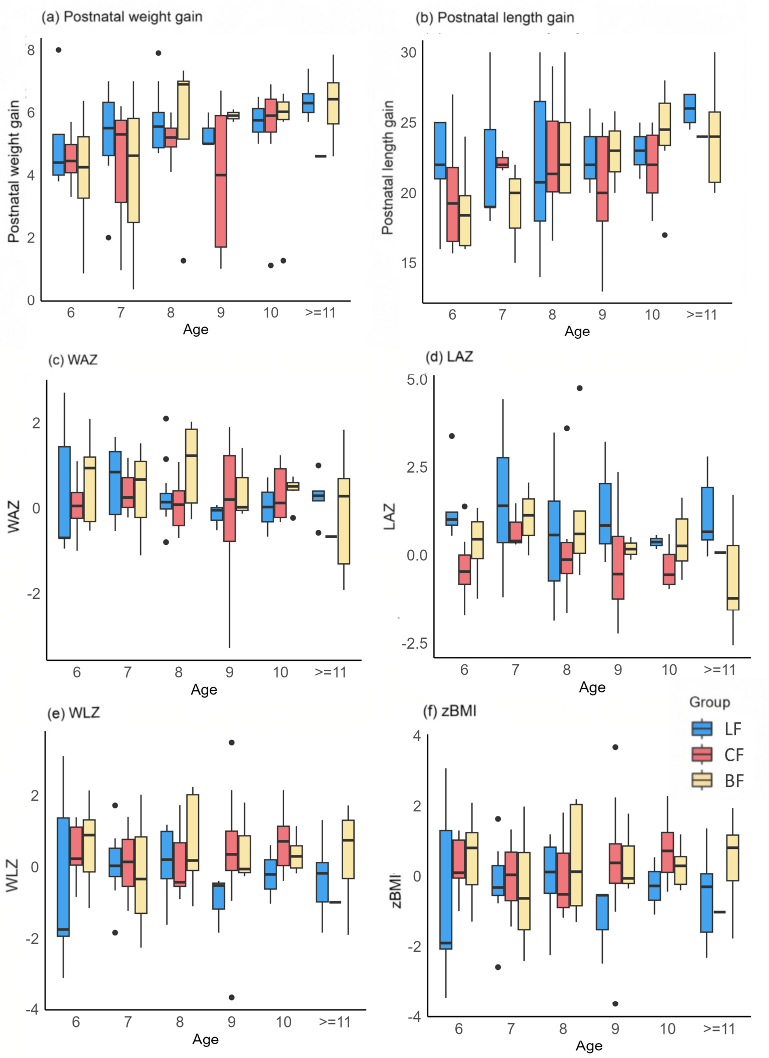

Figure S2. Box plots of post-PSM comparative distributions for (a) postnatal weight gain, (b) postnatal length gain, (c) weight-for-age Z-score (WAZ), (d) length-for-age Z-score (LAZ), (e) weight-for-length Z-score (WLZ), and (f) BMI-for-age Z-score (zBMI)

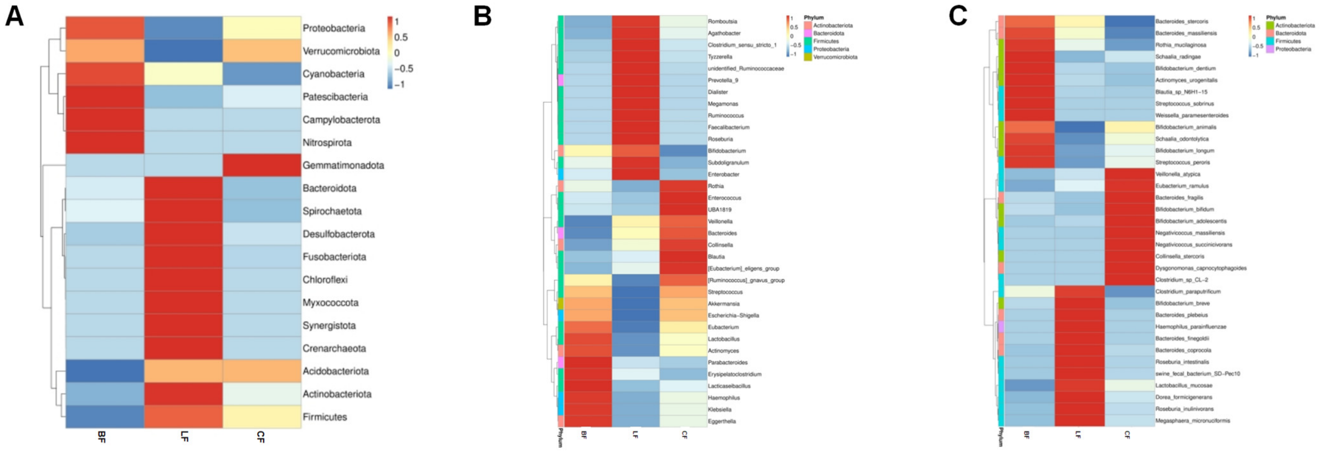

Figure S3. Comparative heat map of infant fecal microbial communities at the level of (A) phylum, (B) genus and (C) species in different feeding groups. LF: Lactoferrin-fortified infant formula; CF: Control formula; BF: Breastfeeding.

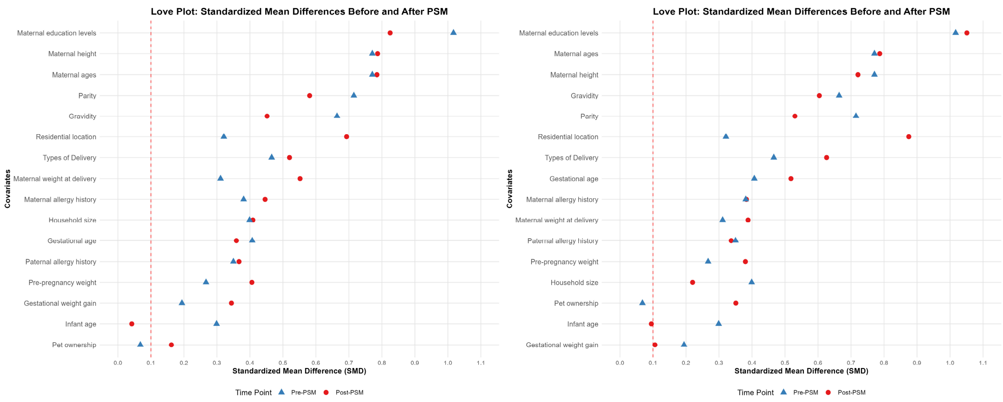

(A) Conducted on infant age and maternal education level

(B) Conducted on all significant imbalanced variables

Figure S4. Love plot for alternative propensity score matching strategies.

Section B

Table S4 Ingredient comparisons of lactoferrin-fortified infant formula (LF) and control formula (CF)

**Item**	**LF formula (100g)**	**CF formula (100g)**
**Energy (kJ)**	2162	2163
**Protein (g)**	10.5	10.5
**Fat (g)**	27.9	27.9
**Linoleic Acid (g)**	4.2	4.3
**α-Linolenic Acid (mg)**	420	430
**Carbohydrates (g)**	55.4	54.6
**Vitamins**		
Vitamin A (μg RE)	385	385
Vitamin D (μg)	12.9	12.9
Vitamin E (α-TE) (mg)	7	7
Vitamin K_1 (μg)	70	70
Vitamin B_1 (μg)	550	550
Vitamin B_2 (μg)	600	600
Vitamin B_6 (μg)	420	420
Vitamin B_{12} (μg)	2.6	2.6
Niacin (Nicotinamide) (μg)	4000	4000
Folic Acid (μg)	79	79
Pantothenic Acid (μg)	2850	2850
Vitamin C (mg)	65	65
Biotin (μg)	16	16
Choline (mg)	130	130
**Minerals**		
Sodium (mg)	190	189.3
Potassium (mg)	420	466
Copper (μg)	386	386.1
Magnesium (mg)	32	32
Iron (mg)	4.75	4.75
Zinc (mg)	3.77	3.77
Manganese (μg)	50	50
Calcium (mg)	350	350
Phosphorus (mg)	220	220
Iodine (μg)	98	98

Item	LF formula (100g)	CF formula (100g)
Chlorine (mg)	325	325
Selenium (μg)	19.5	19.5
**Optional Ingredients**		
Inositol (mg)	/	45
Taurine (mg)	40	40
L-Carnitine (mg)	/	11
Docosahexaenoic Acid (DHA) (mg)	98	120
Arachidonic Acid (ARA) (mg)	110	150
Galactooligosaccharides (GOS) (g)	0.8	1.5
Fructooligosaccharides (FOS) (g)	0.4	1.5
1,3-Dioleoyl-2-palmitoyl Glycerol (OPO) (g)	4.2	6
Nucleotides (mg)	35	35
Lactoferrin (mg)	470	/

Section C

Microbial genomic DNA was extracted from 200 mg of homogenized fecal material, followed by amplification of the V3–V4 hypervariable regions of the bacterial 16S rRNA gene using primers 341F (5′-CCTAYGGGRBGCASCAG-3′) and 806R (5′-GGACTACNNGGGTATCTAAT-3′). Amplified products were purified, and sequencing libraries were constructed through end-repair, adapter ligation, and size selection. Library quality was verified via AATI fragment analysis and QPCR quantification before paired-end sequencing (2 × 250 bp) on the Illumina NovaSeq 6000 platform.

Bioinformatic processing was performed using QIIME2 (v.2022.02): Raw sequences were demultiplexed, trimmed of primers/barcodes, and quality-filtered using `cutadapt v3.3` and `fastp v0.23.1` (parameters: `--min_len 200; -q 20`). High-resolution Amplicon Sequence Variants (ASVs) were generated via DADA2 for denoising and error correction, replacing traditional OTU clustering to enhance taxonomic resolution. Chimeric sequences were filtered using `vsearch v2.16.0`. Taxonomic assignment of ASVs was conducted against the SILVA 138.1 reference database using a pre-trained Naive Bayes classifier (`classify-sklearn`), and a maximum-likelihood phylogenetic tree was constructed with `qiime phylogeny align-to-tree-mafft-fasttree` for downstream beta-diversity analysis.

Table S5 Summary of Data Processing

Sample	RawPE	Combined	Qualified	Nochime	Base(nt)	Avglen(nt)	GC	Q20	Q30
**m39**	149433	148401	146230	137650	56666649	411.67	56.20%	98.20%	94.09%
**m37**	75383	74927	73815	68608	28296257	412.43	56.43%	98.32%	94.37%
**m30**	104903	104468	102952	96770	40718082	420.77	55.07%	98.77%	95.57%
**m40**	149304	148200	145963	136583	56487607	413.58	56.42%	98.23%	94.13%
**m38**	93449	92795	91445	82773	35074698	423.75	56.37%	98.21%	94.04%
**m35**	150650	149557	147686	133860	55710983	416.19	56.19%	98.48%	94.81%
**m33**	117654	116717	114722	107622	44466618	413.17	57.47%	97.99%	93.62%
**m32**	106182	105794	104169	101759	41696790	409.76	57.70%	98.87%	95.91%
**m36**	148463	147723	144743	135814	56026234	412.52	56.39%	98.49%	94.92%
**m34**	142128	141501	138802	134113	54998186	410.09	57.74%	98.79%	95.86%
**m31**	79152	78888	77686	72531	30078415	414.7	56.16%	98.84%	95.81%
**NC01**	102498	102063	100610	87297	36883019	422.5	56.07%	98.88%	95.85%
**m41**	150467	149228	146893	134021	57318334	427.68	56.15%	98.30%	94.21%
**J794**	104417	103996	102440	83937	34715290	413.59	56.45%	98.82%	95.77%
**m15**	105985	105460	103454	93343	40029155	428.84	56.06%	98.66%	95.21%
**m9**	105939	105432	103851	79383	32553215	410.08	54.42%	98.74%	95.48%
**m2**	112690	112263	110424	107659	44392164	412.34	58.12%	98.89%	96.05%
**J811**	102336	101920	100384	77631	32027326	412.56	56.80%	98.84%	95.80%

**m28**	66592	66343	65430	54401	22770165	418.56	57.01%	98.88%	95.90%
**m7**	102392	102039	100490	96582	39944019	413.58	57.32%	98.73%	95.55%
**J693**	102706	102312	100807	94567	38862023	410.95	57.25%	98.89%	95.99%
**J701**	56679	56502	55688	55272	22719966	411.06	57.14%	98.83%	95.80%
**J699**	102793	102406	100907	91179	37468532	410.93	57.23%	98.85%	95.93%
**m11**	102369	101632	99585	83664	35741650	427.2	55.00%	98.51%	94.80%
**CD02**	102336	101916	100432	90566	37119092	409.86	57.12%	98.80%	95.82%
**m14**	106401	105748	104189	102920	43017756	417.97	54.33%	98.73%	95.49%
**m13**	65482	65267	64445	60009	24619949	410.27	57.68%	98.94%	96.12%
**m4**	104776	104317	102613	97632	40896916	418.89	57.62%	98.77%	95.65%
**JY82**	106336	105895	104229	100378	41253228	410.98	57.45%	98.84%	95.90%
**J867**	103617	103219	101641	81444	33238074	408.11	62.78%	98.62%	95.29%
**m3**	104224	103829	102297	96948	39874901	411.3	58.12%	98.72%	95.58%
**m8**	94248	93868	92372	75677	31498988	416.23	55.51%	98.79%	95.63%
**m5**	105449	105056	103283	100861	41367302	410.14	57.66%	98.84%	95.92%
**J46**	102154	101798	100137	91611	37946386	414.21	56.65%	98.70%	95.48%
**J684**	102191	101762	100338	91731	37530314	409.13	57.47%	98.99%	96.33%
**Y1363**	109292	108668	106801	88405	37237527	421.22	55.51%	98.68%	95.35%
**Y1316**	103760	103163	101411	92983	39460470	424.38	53.58%	98.64%	95.29%
**J580**	108683	108061	106431	70064	29174624	416.4	53.44%	98.90%	95.95%
**J522**	104473	104055	102352	95959	39593246	412.61	56.84%	98.71%	95.46%
**J521**	102536	102141	100606	79721	33091089	415.09	56.14%	98.77%	95.64%
**J540**	103724	103366	101715	97099	40008716	412.04	56.33%	98.79%	95.68%
**J36**	106187	105800	104349	89122	36609735	410.78	57.96%	98.93%	96.14%
**J27**	110834	110159	108260	97464	41075520	421.44	52.32%	98.67%	95.27%
**J671**	103117	102631	101312	85982	35215481	409.57	56.74%	99.01%	96.27%
**J239**	102295	101640	99898	60071	25165661	418.93	52.96%	98.73%	95.34%
**J532**	102998	102567	100961	82166	33759023	410.86	56.57%	98.84%	95.89%
**J445**	163678	163050	159603	141573	58657519	414.33	56.54%	98.59%	95.25%
**J322**	104547	103836	101984	81830	34314692	419.34	53.28%	98.60%	95.16%
**J690**	118210	117750	116118	92615	38416141	414.79	56.36%	98.51%	94.85%
**J558**	111052	110397	108415	66685	27132430	406.87	54.93%	98.64%	95.23%
**J531**	105658	105213	103585	76002	31637036	416.27	54.03%	98.81%	95.78%
**J523**	106729	106207	104676	88901	36374591	409.16	55.96%	98.86%	95.91%
**J268**	105618	105218	103785	93025	38294616	411.66	56.64%	98.81%	95.78%
**Y63**	104550	104119	102463	73645	30635350	415.99	56.67%	98.80%	95.74%
**Y1225**	103640	103112	101509	82194	33909899	412.56	55.34%	98.81%	95.71%
**Y103**	106892	106435	104846	90553	37447384	413.54	57.32%	98.78%	95.72%
**Y72**	102974	102483	100738	83924	34490756	410.98	56.84%	98.73%	95.54%
**Y69**	102740	102278	100500	83117	34943513	420.41	55.63%	98.59%	95.18%
**Y437**	114652	114045	111939	94885	40284535	424.56	53.77%	98.62%	95.14%
**Y104**	106268	105760	104295	85056	35878990	421.83	58.06%	98.84%	95.71%
**Y86**	88472	88024	86547	76977	32343145	420.17	53.31%	98.68%	95.34%
**Y82**	113182	112708	111050	96074	40230298	418.74	56.09%	98.93%	96.02%

**Y75**	105628	105192	103792	88651	36333899	409.85	58.25%	99.01%	96.35%
**Y54**	105608	105262	103731	91145	37480855	411.22	57.30%	98.67%	95.37%
**Y101**	103050	102573	100938	80264	32901762	409.92	56.69%	98.85%	95.93%
**Y1191**	103231	102800	101246	64362	26289001	408.46	55.50%	98.90%	96.00%
**Y48**	104218	103932	102396	97460	40209338	412.57	58.74%	98.95%	96.13%
**Y88**	113618	113163	111436	97661	40092967	410.53	59.34%	98.85%	95.92%
**Y87**	104136	103700	101949	88057	36672120	416.46	56.46%	98.79%	95.66%
**Y73**	106589	106027	104420	90231	37892905	419.95	55.29%	98.86%	95.81%
**Y99**	116616	116005	114239	103398	42681354	412.79	59.02%	98.89%	95.99%
**Y95**	106548	106100	104665	89608	36578160	408.2	60.91%	98.92%	96.13%
**Y85**	116360	115867	114171	102737	42500226	413.68	57.10%	98.68%	95.47%
**Y66**	106327	105886	104330	81625	33996182	416.49	55.94%	98.96%	96.18%
**Y13**	106033	105662	104048	91535	37507795	409.76	61.73%	98.86%	95.85%
**Y37**	114965	114525	112545	96517	40166265	416.16	56.60%	98.67%	95.40%
**Y21**	103207	102753	101051	83878	34974253	416.97	55.79%	98.70%	95.45%
**Y31**	104357	103877	102001	85181	35105700	412.13	60.74%	98.66%	95.35%
**Y40**	61485	61261	60343	51608	21139858	409.62	56.21%	98.89%	95.96%
**Y32**	103743	103151	101364	72373	30233636	417.75	55.53%	98.66%	95.29%
**Y29**	68839	68642	67763	52396	21715687	414.45	57.25%	98.89%	95.94%
